# Genetic Analysis for Cooking and Eating Quality of Super Rice and Fine Mapping of a Novel Locus *qGC10* for Gel Consistency

**DOI:** 10.3389/fpls.2020.00342

**Published:** 2020-03-24

**Authors:** Anpeng Zhang, Yang Gao, Yuanyuan Li, Banpu Ruan, Shenglong Yang, Chaolei Liu, Bin Zhang, Hongzhen Jiang, Guonan Fang, Shilin Ding, Noushin Jahan, Lihong Xie, Guojun Dong, Zhengjin Xu, Zhenyu Gao, Longbiao Guo, Qian Qian

**Affiliations:** ^1^Rice Research Institute of Shenyang Agricultural University/Key Laboratory of Northern Japonica Rice Genetics and Breeding, Ministry of Education and Liaoning Province, Shenyang, China; ^2^State Key Laboratory of Rice Biology, China National Rice Research Institute, Hangzhou, China

**Keywords:** cooking and eating quality, gel consistency, QTL analysis, fine mapping, rice

## Abstract

Rice (*Oryza sativa* L.) is an important cereal that provides food for more than half of the world’s population. Besides grain yield, improving grain quality is also essential to rice breeders. Amylose content (AC), gelatinization temperature (GT) and gel consistency (GC) are considered to be three indicators for cooking and eating quality in rice. Using a genetic map of RILs derived from the super rice Liang-You-Pei-Jiu with high-density SNPs, we detected 3 QTLs for AC, 3 QTLs for GT, and 8 QTLs for GC on chromosomes 3, 4, 5, 6, 10, and 12. *Wx* locus, an important determinator for AC and GC, resided in one QTL cluster for AC and GC, *qAC6* and *qGC6* here. And a novel major QTL *qGC10* on chromosome 10 was identified in both Lingshui and Hangzhou. With the BC_4_F_2_ population derived from a CSSL harboring the segment for *qGC10* from 93-11 in PA64s background, it was fine mapped between two molecular markers within 181 kb region with 27 annotated genes. Quantitative real-time PCR results showed that eight genes were differentially expressed in endosperm of two parents. After DNA sequencing, only *LOC_Os10g04900*, which encodes a F-box domain containing protein, has 2 bp deletion in the exon of PA64s, resulting in a premature stop codon. Therefore, *LOC_Os10g04900* is considered to be the most likely candidate gene for *qGC10* associated with gel consistency. Identification of *qGC10* provides a new genetic resource for improvement of rice quality.

## Introduction

Rice is one of the most important crops served as staple food for more than half of world population. Rice planting area has reached 30.18 million hectares in 2018, and rice production was about 202.70 million tons, accounting for 25.80% of total grain output. It is a milestone in the history of rice breeding that the application of hybrid rice in 1970s (Li et al., 2016). The development of hybrid rice in China has made great contribution to the world’s rice yield. Recently, besides pursuing of high yield, more attention has been paid to rice quality. Liang-You-Pei-Jiu, the super hybrid rice from the cross of the *indica* variety 93-11 and the light-thermo-sensitive genic male sterile line PA64s was a successful example with 10.5 tons per hectare in 2000 (Gao et al., 2013). However, compared to rice production, the development of quality breeding was relatively stunted (Su et al., 2011). Therefore, with better living conditions, in order to meet the demands of people, rice quality needs to be improved by breeders.

Rice quality includes various characteristics when it is being produced, processed, sold, cooked and ate. In general, it involves processing quality (also called milling quality), appearance quality, cooking and eating quality and nutritional quality. High quality rice included 14 indicators, among which the milled rice rate, chalky degree, eating quality and amylose content were grading indexes while roughness, chalky rate, grain shape, gel consistency were indexes for reference. The amylose content (AC), gelatinization temperature (GT) and gel consistency (GC) are three major indicators for physical and chemical characteristics of the starch in rice endosperm which, directly affected cooking and eating quality (Sun S. -Y. et al., 2005).

GC, being a kind of colloidal property of rice starch and referring to viscosity of 4.4% rice glue, is an important indicator of rice quality. It can be divided into three categories: soft (>60 mm), adhesive (40–60 mm) and hard GC (<40 mm). Rice with hard GC is rough and fluffy, and becomes hard and dry after cool; while rice with soft GC is soft and elastic, and still keeps soft after cooling. Usually, the larger GC, the softer, stickier, better taste and shinier rice (Wang et al., 2007).

Previous studies indicated natural genetic variations for rice quality (Tian et al., 2009). As a quantitative trait, GC is suitable for QTL analysis and its genetic basis is also complicated. So far, 22 QTLs for GC have been reported on chromosomes 1, 2, 3, 6, 7, and 10^1^. He et al. (1999) detected two major QTLs for GC on chromosomes 2 and 7, which explain 20.2 and 14.2% of the genetic variation, respectively. Huang et al. (2000) used rice varieties with high GC and middle GC as two parents to construct the recombinant inbred lines (RILs), and detected two major QTLs for GC on chromosome 3. A major QTL for GC was identified by State Key Laboratory of Rice Biology, China National Rice Research Institute with double haploid (DH) population came from TN1 and CJ6, and further fine mapped and confirmed to be the *Wx* gene (Su et al., 2011). Because the *Wx* gene was reported to controlling AC, and GC was found negatively correlated with AC (Zhang et al., 2002; Sun Y. et al., 2005), therefore, both GC and AC were controlled by *Wx*. However, association analysis showed the *Wx* gene only account for nearly 40% of phenotypic variation for GC (Tian et al., 2009). And some varieties with same level of AC exhibited different GC (Sun Y. et al., 2005). A gene responsible for GT, *ALK*, was also reported as a modifier gene for GC in rice (Gao et al., 2011). Thus, other QTLs/genes are to be mapped and isolated to clarify the genetic basis for GC.

Although QTL mapping for rice quality initiated in the 1990s, few studies on fine mapping of QTLs for cooking and eating quality have been reported. Here, we detected QTLs for cooking and eating quality of super rice and fine mapped a novel QTL *qGC10* for GC, which will help further cloning of the QTL/gene and improvement of rice quality.

## Materials and Methods

### Cultivation and Management of Experimental Materials

A RIL from a cross between 93-11 and PA64s were used in this study for cooking quality traits analysis (Gao et al., 2013). Total of 1779 high-quality polymorphic SNP markers were used to construct the linkage map with 1568.21 cM (Supplementary Data S1). The parents 93-11 is an indica variety and the PA64s is the indica type light-thermo sensitive genic male sterile line. A total of 116 and 102 RILs were cultivated in Hangzhou (HZ) in 2011 and Lingshui (LS) in 2011, 2012, respectively. Each line was grown in triplicate in a randomized block design. The plot size was four rows of six plants with 20 × 20 cm spacing. Field management and chemical input for disease and pest control followed the standard procedures to avoid yield loss during the whole growth duration. Mature seeds of each line (3 biological repetitions) and two parents (6 biological repetitions) were harvested 30 days after heading and dried in an electro-thermal incubator (ZXDP-A2160, Shanghai) at 30°C for 72 h. The dried seeds (20 g) were shucked and polished.

### Development of Fine Mapping Population

To develop a chromosome segment substitution line (CSSL) containing the *qGC10* for GC detected both in Lingshui and Hangzhou on chromosome 10, a line of RILs with 93-11 genotype in the *qGC10* region was selected to backcross with recurrent parent PA64s. Two markers IND-1 and IND-6 (Supplementary Table S1) were used for marker assisted selection (MAS) of each generation. As a result, a BC_4_F_1_ line was constructed exhibiting heterozygous across the entire *qGC10* region with genetic background of PA64s. After self-crossing, a BC_4_F_2_ population was obtained for fine mapping of *qGC10*. A NIL carrying homozygous allele of 93-11 in the target QTL region between markers IND-4 and SNP-1 (Supplementary Table S1), designated NIL-*qGC10*, was also developed with PA64s background.

### Measurement of Cooking and Eating Quality in Rice

Apparant Amylose content (AAC) was measured following the procedure of Perez and Juliano with some modifications (Perez and Juliano, 1978). Absorbance of the starch solution was determined at 620 nm using the spectrophotometer. Gelatinization temperature (GT) was indirectly estimated via the alkali digestion test (Little et al., 1958). Six whole-grain, milled rice samples were placed in triplicate square plastic boxes containing 10 mL 1.7% potassium hydroxide (KOH). The boxes were incubated for 23 h at 30°C. Grain appearance and disintegration were visually rated after incubation based on the standard numerical scale and expressed with alkali spreading value (ASV). Gel Consistency (GC) was evaluated according to Cagampang et al. (1973). Rice starch becomes rice paste glue after dilute alkali and heat treatment, after cooling in the horizontal tube has a certain degree of extension (Tran et al., 2011). Technical measurements were performed for each sample in triplicate.

### DNA Isolation and PCR Analysis

Genomic DNA of the parents and BC_4_F_2_ individuals was extracted from fresh leaves using the CTAB method (Aline et al., 2009). DNA amplification was performed using a Gene Amp PCR system 9700 thermo cycler. PCR was performed in a 15 μL reaction mix including 25 ng genomic DNA, 2 μL of each primer, 1.0 μL 10 × PCR buffer, 0.1 mmol/L dNTP, 0.2 μL 5 U/μL Taq DNA polymerase (Tiangen Biotech, Beijing, China) and 1.5 μL ddH2O. Amplification conditions consisted of an initial denaturation at 94°C for 5 min, 40 cycles of 94°C for 30 s, 55–60°C for 30 s, and 72°C for 30 s, followed by a final extension at 72°C for 10 min, and saving at 15°C forever.

### Statistical Analyses and QTL Analysis

All statistical analyses were completed using the SAS (Statistical Analysis System) v8.01. QTL analysis was performed with the MultiQTL package^2^ using the maximum likelihood interval mapping approach for the RIL-selfing population. For major-effect QTLs, the LOD threshold was obtained based on a permutation test (1000 permutations, *P* = 0.05) for each dataset. QTLs were named according to McCouch et al. (1997).

### Design of Markers for Fine Mapping

Primers were designed in *qGC10* region on the basis of insertions/deletions (InDels) and SNPs identified between 93-11 and PA64s (Supplementary Table S1) (Gao et al., 2013). Genotypes of SNP markers were screened by high-resolution dissociation curve analysis system (LightScanner 96, Idaho Technology Inc.).

### Determination of RVA

Pasting properties of starch granules were analyzed by RVA model 3-D (Newport Scientific, Sydney, NSW, Australia), a programmed heating and cooling cycle was followed, as described by Toyoshima et al. (1997) and analyzed with Thermal Cycle for Windows (TCW). Duplicate measurements were performed for each sample of each line (3 biological repetitions). The main steps and the relevant parameters: Three gram flour of each sample at 14% moisture was weighed into aluminum canister, then 25 mL distilled water was added. A paddle was placed in the canister to mix the sample. The RVA disperses the samples by rotating the paddle at 960 rpm for the first l0 s of the test, after which the viscosity is sensed using a constant paddle rotation speed of 160 rpm. The idle temperature is set to 50°C and the following 12.5 min test profiles were run: (1) 50°C held for 1.0 min, (2) the temperature is linearly ramped up to 95°C until 4.8 min, (3) the temperature is held at 95°C until 7.5 min, (4) the temperature is linearly ramped down to 50°C at 11 min, (5) held at 50°C until 12.5 min (Yan et al., 2005). The determination was repeated two times per sample. RVA curve was described by six parameters: peak viscosity, holding strength, breakdown, final viscosity, consistence and setback. All the viscosity parameters were expressed in Rapid Viscosity Units (RVU).

## Results

### Phenotypic Variation of Cooking and Eating Quality in the Parents and RILs

**FIGURE 1:**
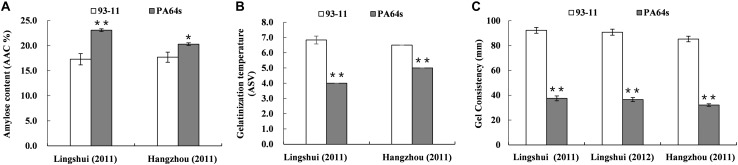
Phenotype of amylose content (AAC, **A**), gelatinization temperature (ASV, **B**) and gel consistency **(C)** in parents 93-11 and PA64s. The error bar for each value represents mean ± SD (*n* = 6). * and ** indicate at 5 and 1% significant level compared to 93-11 according to *t-*test (*n* = 6), respectively.

Significant differences existed in AAC, GT and GC between two parents in both Lingshui and Hangzhou (Table 1 and Figure 1). A total of 116 and 102 RILs, together with their parents from Hangzhou and Lingshui were investigated respectively for the three indexes for cooking and eating quality. For each trait, the distribution of the RIL population appeared continuously with transgressive characteristic in the two environments. Nearly normal continuous distributions of phenotypic values were observed from LS population for AAC and GC and HZ population for GC, which indicated that AAC and GC were controlled by poly-genes (Supplementary Figure S1). In HZ population, AAC and GT showed bimodal distribution of phenotypic values, which indicated that AAC and GT were controlled by a major gene and some minor genes (Supplementary Figure S1).

**TABLE 1:** Phenotypic variation of cooking and eating quality traits in the parents.

Harvested location	Traits	Parents		RIL population		
		93-11	PA64s	Mean ± SD	Minimum	Maximum
Lingshui (2011)	AAC (%)	17.29 ± 1.01	23.09 ± 0.26**	19.41 ± 3.76	13.53	28.82
	GT (ASV)	6.8 ± 0.3	4.0 ± 0.0**	6.57 ± 0.69	4.3	7.0
	GC (mm)	92.30 ± 2.37	37.50 ± 2.00**	80.65 ± 14.02	28.00	100.00
(2012)	GC (mm)	90.83 ± 2.44	36.50 ± 1.66**	82.28 ± 13.32	28.00	100.00
Hangzhou (2011)	AAC (%)	17.70 ± 1.12	20.30 ± 0.28*	18.27 ± 6.05	4.36	30.82
	GT (ASV)	6.5 ± 0.0	5.0 ± 0.0**	5.68 ± 1.33	3.0	7.0
	GC (mm)	85.30 ± 2.25	32.10 ± 1.01**	65.96 ± 13.25	30.5	99

AAC, apparant amylose content; GT, gelatinization temperature; GC, gel consistency. Mean ± SD. * and ** indicate at 5 and 1% significant level compared to 93-11 according to t-test (n = 6), respectively.

### Correlation Analysis of Three Traits

Based on Pearson’s correlation analysis, trait correlations between cooking and eating quality parameters showed that GC exhibited significantly negative correlation to AAC and GT in HZ and GT in LS (Table 2). The correlation between AAC and GC was negative and highly significant in HZ while little in LS. Similarly, GC showed a significantly negative correlation to GT in both LS and HZ. There was no significant correlation between AAC and GT, whether in LS or HZ. These findings supported that GC is an important factor affecting cooking and eating quality.

**TABLE 2:** Correlation coefficients between AC, GT, and GC.

	AAC vs. GT	AAC vs. GC	GT vs. GC
Lingshui (2011)	0.101	−0.035	−0.264*
Hangzhou (2011)	0.032	−0.314**	−0.213*

AAC, apparant amylose content; GT, gelatinization temperature; GC, gel consistency. * and ** indicate at 5 and 1% significant level, respectively.

### Identification of QTLs for Cooking and Eating Quality Traits

**FIGURE 2:**
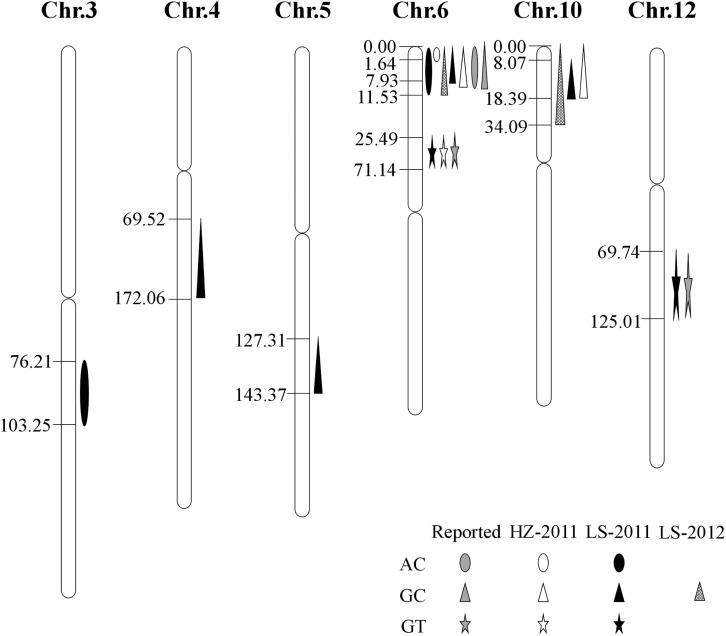
Location of QTLs for AC, GT, and GC on SNP map. Number indicates genetic distance (cM) along each chromosome.

A linkage map with high density SNP markers covering 12 chromosomes has been established to detect QTLs for rice cooking and eating quality traits, with a total distance of 1568.21 cM and the average distance of about 0.88 cM between the markers. A total of 14 QTLs, including 3 QTLs for AC, 3 QTLs for GT and 8 QTLs for GC were identified on chromosomes 3, 4, 5, 6, 10, and 12 (Table 3 and Figure 2). One QTL cluster for AC and GC was detected on chromosome 6. The *qAC6* covering the *Wx* gene (Fan et al., 2005), detected in both LS and HZ between markers SNP6-1 and SNP6-27, SNP6-1 and SNP6-11 on chromosome 6, explained 35.7 and 64.9% of phenotypic variation, respectively. Meanwhile, the *qGT6* in LS and HZ covering the *ALK* gene (Gao et al., 2011), were identically mapped between SNP6-50 and SNP6-96 on chromosome 6, explained 23.4 and 41.8% of total phenotypic variation in LS and HZ, respectively. The major QTL, *qGC6*, was preliminary mapped on chromosome 6 in the interval of SNP6-1 and SNP6-19 in LS in 2011, SNP6-1 and SNP6-27 in LS in 2012, SNP6-1 and SNP6-23 in HZ in 2011 accounting for 11.4, 12.3, and 11.2% of phenotypic variance, individually. The *qGC10* was identified on chromosome 10 for three times with explained phenotypic variation of 13.8, 14.0, and 12.2%, respectively in LS in 2011, 2012 and HZ in 2011.

**TABLE 3:** QTLs for AC, GT, and GC in the RIL population.

Trait	Chromosome	LOD	Genetic position (cM)	*SE*	PEV (%)	Marker
AC-LS-2011	3	3.6	76.21–103.25	−2.00	2.4	SNP3-191∼SNP3-273
AC-LS-2011	6	19.8	0.00–11.53	−4.77	35.7	SNP6-1∼SNP6-27
AC-HZ-2011	6	33.3	0.00–1.64	−6.59	64.9	SNP6-1∼SNP6-11
GC-LS-2011	4	2.6	69.52–172.06	−7.27	6.4	SNP4-130∼SNP4-261
GC-LS-2011	5	3.0	127.31–143.37	8.03	7.9	SNP5-142∼SNP5-157
GC-LS-2011	6	3.4	0.00–7.93	9.68	11.4	SNP6-1∼SNP6-19
GC-LS-2011	10	3.8	8.07–18.39	10.63	13.8	SNP10-9∼SNP10-21
GC-LS-2012	6	3.3	0.00–11.53	9.26	12.3	SNP6-1∼SNP6-27
GC-LS-2012	10	3.9	0.00–34.09	9.88	14.0	SNP10-1∼SNP10-36
GC-HZ-2011	6	3.2	0.00–9.52	9.27	11.2	SNP6-1∼SNP6-23
GC-HZ-2011	10	2.5	0.00–18.39	9.08	12.2	SNP10-1∼SNP10-21
GT-LS-2011	6	7.6	25.49–71.14	1.27	23.4	SNP6-50∼SNP6-96
GT-LS-2011	12	3.1	69.74–125.01	0.93	12.6	SNP12-95∼SNP12-143
GT-HZ-2011	6	10.6	25.49–71.14	0.84	41.8	SNP6-50∼SNP6-96

### The Relationship Between Rapid Visco Analyzer (RVA) Profile Characteristic Values and GC

**FIGURE 3:**
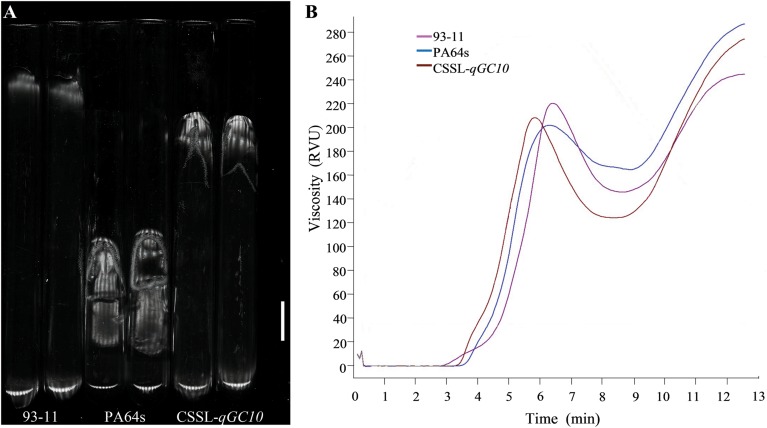
GC phenotype and Rapid Visco Analyzer (RVA) profile characteristic values in 93-11, PA64s and CSSL-*qGC10*. **(A)** GC phenotype in 93-11, PA64s and CSSL-*qGC10*. Rice flour becomes rice paste glue after dilute alkali and heat treatment. It has a certain degree of extension when cooling in the horizontal tube with total length of 100 mm, and the extended length represents GC value. **(B)** RVA profile characteristic values in 93-11, PA64s and CSSL-*qGC10*. The RVA spectrum simulates the process of rice cooking and reflects the taste and texture of rice. RVA is represented by Relative Viscosity Units (RVU).

The determination of rice viscosity can simulate the dynamic changes of the rice cooking process, and the viscosity characteristics are measured to reflect the taste and texture of the rice. GC is an important index that affects the RVA profile characteristic values (Jia et al., 2008). Therefore, we determined the RVA profiles of two parents and CSSL-*qGC10*. Consistent with GC phenotype (Figure 3A), RVA spectrum of PA64s showed the lowest peak viscosity and the highest final viscosity, indicating that the cold rice after cooking may become harder (Table 4 and Figure 3B). Peak viscosity and final viscosity of CSSL-*qGC10* exhibited values between 93-11 and PA64s. Meanwhile, PA64s had the smallest breakdown value and the largest setback value, which is in agreement with the previous reports (Zheng et al., 2018).

**TABLE 4:** Rapid Visco Analyzer (RVA) profile characteristic values in 93-11, PA64s and CSSL-*qGC10.*

Variety/Line	Peak viscosity	Holding strength	Final viscosity	Breakdown	Consistency	Setback
93-11	222.17 ± 1.65^a^	145.09 ± 1.65^b^	245.33 ± 0.35^b^	77.09 ± 3.30^a^	100.25 ± 1.29^c^	23.17 ± 2.00^c^
PA64s	203.29 ± 1.12^c^	166.54 ± 1.82^a^	288.00 ± 0.59^a^	36.75 ± 0.71^b^	121.46 ± 1.23^b^	84.71 ± 0.53^a^
CSSL-*qGC10*	208.25 ± 0.95^b^	126.92 ± 3.30^c^	269.79 ± 7.48^a^	81.33 ± 4.24^a^	142.88 ± 10.78^a^	61.55 ± 6.54^b^

*Breakdown is equal to the difference between peak viscosity and holding strength, Consistency is equal to the difference between final viscosity and holding strength, Setback is equal to the difference between final viscosity and peak viscosity. Data are means (SD) and duplicate measurements were performed for each sample (n = 3). Different superscript letters represent significant differences (P < 0.05) by t-test.*

### Fine Mapping of *qGC10*

**FIGURE 4:**
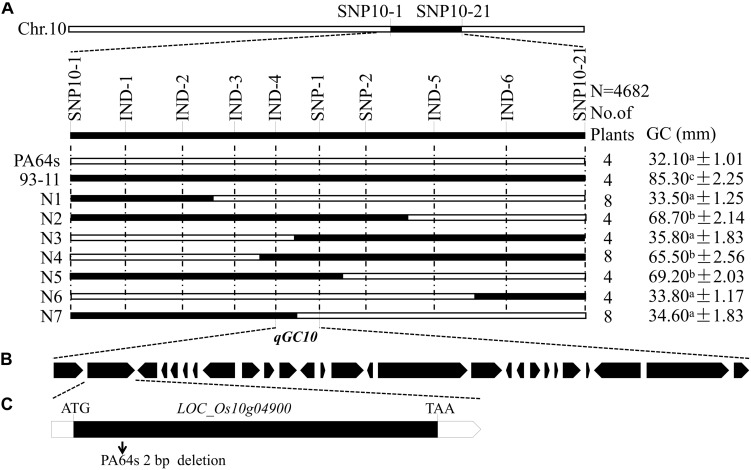
Fine mapping of *qGC10*. **(A)** *qGC10* was narrowed down to a 181 kb interval defined by markers IND-4 and SNP-1. Values represent means ± SD (*n* ≥ 3). Different letters represent significant differences (*P* < 0.05) by *t*-test. **(B)** All the 27 predicted genes in the target region. **(C)** Structure and mutated sites of the most likely candidate gene. *LOC_Os10g04900* consist of one exon (represent with black box). Arrow indicates the deletion of 2 bp in the position of PA64s compared to 93-11.

For fine mapping of *qGC10*, a CSSL CSSL*-qGC10* with PA64s background was constructed to develop BC_4_F_2_ population by backcrossing to PA64s and self-pollination. Based on the resequencing data of 93-11 and PA64s, several InDel markers were designed to screen the population (Supplementary Table S1). According to the GC value, lines with high GC (>60 mm) and low GC (¡40 mm) were selected for fine mapping. Combining the phenotype and genotype analysis, *qGC10* was finally delimited in 181 kb region between markers IND-4 and SNP-1 on chromosome 10 (Figure 4A).

### Candidate Genes Determination at the *qGC10* Locus

**FIGURE 5:**
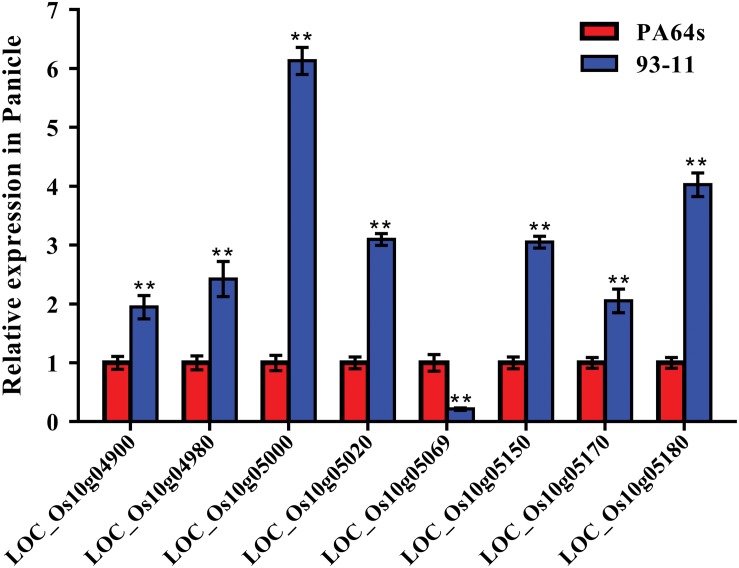
Relative expression of 8 genes in the endosperm of 93-11 and PA64s. The error bar for each value represents mean ± SD (*n* = 3). ** indicate at 1% significant level compared to PA64s according to *t* test.

According to Rice Genome Annotation Project Database^3^, there were 27 annotated genes in 181 kb target region for *qGC10* on chromosome 10 (Table 5 and Figure 4B). Among them, 8 genes differentially expressed in endosperm between 93-11 and PA64s were sequenced and 2 bp deletion (G_84_G_85_) was detected in the exon of *LOC_Os10g04900* in PA64s (Figures 4C, 5 and Supplementary Figure S2), resulting in a premature stop codon and a protein with only 28 amino acids. And *LOC_Os10g04900* is highly expressed in the panicle (Supplementary Figure S3). Therefore, *LOC_Os10g04900* was considered the most likely candidate gene for *qGC10* locus associated with GC.

**TABLE 5:** Candidate genes at the *qGC10* locus.

Gene ID	Annotation
*LOC_Os10g04890*	Expressed protein
*LOC_Os10g04900*	OsFBX364 - F-box domain containing protein, expressed
*LOC_Os10g04910*	Expressed protein
*LOC_Os10g04930*	Expressed protein
*LOC_Os10g04940*	Hypothetical protein
*LOC_Os10g04950*	Hypothetical protein
*LOC_Os10g04960*	Expressed protein
*LOC_Os10g04980*	OsFBX365 - F-box domain containing protein, expressed
*LOC_Os10g04990*	Expressed protein
*LOC_Os10g05000*	OsFBX366 - F-box domain containing protein, expressed
*LOC_Os10g05010*	Expressed protein
*LOC_Os10g05020*	Cytochrome P450, putative, expressed
*LOC_Os10g05030*	Expressed protein
*LOC_Os10g05040*	Transposon protein, putative, CACTA, En/Spm sub-class, expressed
*LOC_Os10g05050*	Expressed protein
*LOC_Os10g05069*	Lysosomal alpha-mannosidase precursor, putative, expressed
*LOC_Os10g05088*	GDSL-like lipase/acylhydrolase, putative, expressed
*LOC_Os10g05110*	Retrotransposon protein, putative, unclassified
*LOC_Os10g05120*	Retrotransposon protein, putative, unclassified, expressed
*LOC_Os10g05130*	Expressed protein
*LOC_Os10g05140*	Hypothetical protein
*LOC_Os10g05150*	KAZ2 - Kazal-type serine protease inhibitor precursor, putative, expressed
*LOC_Os10g05160*	Expressed protein
*LOC_Os10g05170*	OsWAK100 - OsWAK receptor-like cytoplasmic kinase OsWAK-RLCK, expressed
*LOC_Os10g05180*	26S proteasome regulatory subunit S5A, putative, expressed
*LOC_Os10g05190*	Transposon protein, putative, CACTA, En/Spm sub-class, expressed
*LOC_Os10g05200*	OsFBX367 - F-box domain containing protein, expressed

## Discussion

High density genetic linkage map combined with different populations facilitate QTL identification and fine mapping. With different genetic populations, such as F_2_ population, double haploid (DH) population, RILs, CSSLs and near-isogenic lines (NILs), hundreds of genomic regions associated with cooking and eating quality traits have been identified in rice (Su et al., 2011). Among them, CSSLs and NILs are effective materials for QTL mapping and positional cloning owing to all lines have the same background without complex variations in genetic background (Zhou et al., 2009; Anis et al., 2019).

AC, GT, and GC are considered to be three vital traits that determine cooking and eating quality of rice (Tan et al., 1999). As quantitative traits, they are determined mainly by genetic factors and affected by environmental conditions (Fan et al., 2005). Series of QTLs have been reported for AC, GT and GC (Sun S. -Y. et al., 2005; Su et al., 2011; Ponce et al., 2018; Yang et al., 2018). However, only few QTLs have been fine mapped or cloned. In the study, we used a RIL population derived from a cross between PA64s and 93-11 to identify QTLs for AC, GT under two environments, and GC under three environments. Some QTLs were environmentally dependent, such as *qAC3*, *qGT12*, *qGC4* and *qGC5* detected in LS in 2011, whose phenotypic variances explained (PEV) were relatively lower. However, stable QTLs detected in different environments, for example, a novel major QTL *qGC10* detected in both LS (2011 and 2012) and HZ (2011) were suitable for fine mapping further.

In Hangzhou, GC showed negative but significant correlation with AC (Table 2), which indicated that AC affect GC in cooked rice. Both in Lingshui and Hangzhou, GC was also negatively correlated with GT. These results suggested glossy sticky rice texture tend to have high AC and undergo fast breakdown of starch molecules (Su et al., 2011; Pang et al., 2016), supporting the fact that indeed GC was the important factor affecting cooking quality traits (Bao et al., 2006; Gao et al., 2011).

Notably, chromosome 6 contained most of QTLs identified here responsible for AC, GT and GC, where located one QTL cluster for AC and GC. We also detected a major QTL for AC, *qAC6*, located on the same locus of previously reported QTL on chromosome 6 (Sabouri et al., 2012). AC was related with variations at the *waxy* gene on chromosome 6 and several other modifiers (Lanceras et al., 2000; Septiningsih et al., 2003; Aluko et al., 2004; Takeuchi et al., 2007). GT was controlled by a major QTL on chromosome 6 covering the *ALK* gene (He et al., 1999; Lanceras et al., 2000; Umemoto et al., 2002; Gao et al., 2003), where the *qGT6* mapped here. The *qGT12* for GT identified on chromosome 12 in our study was also reported previously (Liu et al., 2014). Therefore, our results were consistent with previous studies that GT was controlled by *ALK* and other minor QTLs.

Several major QTLs for GC have been reported, such as *qGC6*, whose mapped interval including the *Wx* gene. Previous studies showed that *Wx* also regulated GC in rice, and in turn affected grain quality (Su et al., 2011). In our experiments, one QTL for GC, *qGC6* detected on chromosome 6, was co-located with *qAC6*. This QTL cluster covered the *Wx* gene, which play an important role in controlling AC. *Wx* is not only the key gene for AC, but also a modifier gene for GC in rice (Tian et al., 2009). Besides, a novel major QTL, *qGC10* for GC was mapped on chromosome 10. We further fine mapped the *qGC10* within 2.37–2.55 Mb region with a BC_4_F_2_ population. In the target region, 8 genes differentially expressed in endosperm were sequenced and only *LOC_Os10g04900*, encoding a F-box domain containing protein, was found to have 2 bp insertion/deletion in the exon between two parents, 93-11 and PA64s (Figures 4C, 5 and Supplementary Figure S2). Quantitative real-time PCR results showed that *LOC_Os10g04900* is highly expressed in the panicle (Supplementary Figure S3). Previous study have found that *LOC_Os10g04900* was expressed in the milk stage grains by analyzing expression pattern in different developmental stages in rice (Yang et al., 2012). In other crops, its homologous proteins were also found expressed in seeds of Barley (*Hordeum vulgare*, BAK07103) and Sorghum (*Sorghum bicolor*, XP_002461331)^4^. Hence, *LOC_Os10g04900* is considered the most likely candidate gene for the *qGC10* locus associated with gel consistency and to be confirmed further by complementation test.

## Conclusion

Cooking and eating quality is an economically vital trait for rice. Understanding of genetic mechanism underlying cooking and eating quality will be beneficial for breeding of new rice varieties with high yield and good quality. Total of 3 QTLs for AC, 3 QTLs for GT and 8 QTLs for GC were detected on chromosomes 3, 4, 5, 6, 10, and 12 using high-resolution genetic map of RILs derived from super rice Liang-You-Pei-Jiu. A novel major QTL for GC, *qGC10* was identified on chromosome 10 in rice. With the BC_4_F_2_ population derived from the CSSL-*qGC10*, it was delimited to 181 kb region. The target region contained 27 annotated genes according to Rice Genome Annotation Project Website^5^, among which 8 genes differentially expressed in endosperm were sequenced and only *LOC_Os10g04900* had 2 bp deletion in exon in PA64s, resulting in a premature stop codon. Therefore, *LOC_Os10g04900* is considered the most likely candidate gene for *qGC10* associated with gel consistency. Identification of *qGC10* not only promotes exploration of molecular and biological function of the QTL/gene, but provides a new genetic resource for rice quality improvement.

## Data Availability Statement

The data for this study can be found at: For PA64s genome sequence, the accession number is PRJNA208877 and the link: https://www.ncbi.nlm.nih.gov/bioproject/PRJNA208877.

## Author Contributions

ZG designed the research. AZ, YG, YL, BR, SY, CL, BZ, HJ, GF, SD, NJ, LX, and GD performed the research. AZ, ZX, LG, QQ, and ZG analyzed the data. AZ and ZG wrote the manuscript. All authors read and approved the manuscript.

## Conflict of Interest

The authors declare that the research was conducted in the absence of any commercial or financial relationships that could be construed as a potential conflict of interest.

## Footnotes

1 http://archive.gramene.org/db/qtl/

2 www.multiqtl.com

3 http://rice.plantbiology.msu.edu/cgi-bin/gbrowse/rice/

4 https://www.uniprot.org

5 http://rice.plantbiology.msu.edu/

## Supplementary Material

FIGURE S1 Phenotype distribution of AC, GT, and GC in RIL population in Lingshui (LS) and Hangzhou (HZ). Triplicate measurements were performed for each sample (*n* = 3). Arrowheads indicate the means of 93-11 and PA64s (*n* = 6).

FIGURE S2 Sequence difference of *LOC_Os10g04900* between 93-11 and PA64s.

FIGURE S3 Relative Expression of *LOC_Os10g04900* in different tissues of PA64s. The error bar for each value represents mean ± SD (*n* = 3).

TABLE S1 Primers for fine mapping of *qGC10*.

DATA S1 Statistic summary of genetic map.
