# Supplementary figures and images for: Genetic Analysis for Cooking and Eating Quality of Super Rice and Fine Mapping of a Novel Locus qGC10 for Gel Consistency
Source: Front Plant Sci. 2020 Mar 24;11:342. doi: 10.3389/fpls.2020.00342 (PMC7105826; doi:10.3389/fpls.2020.00342)

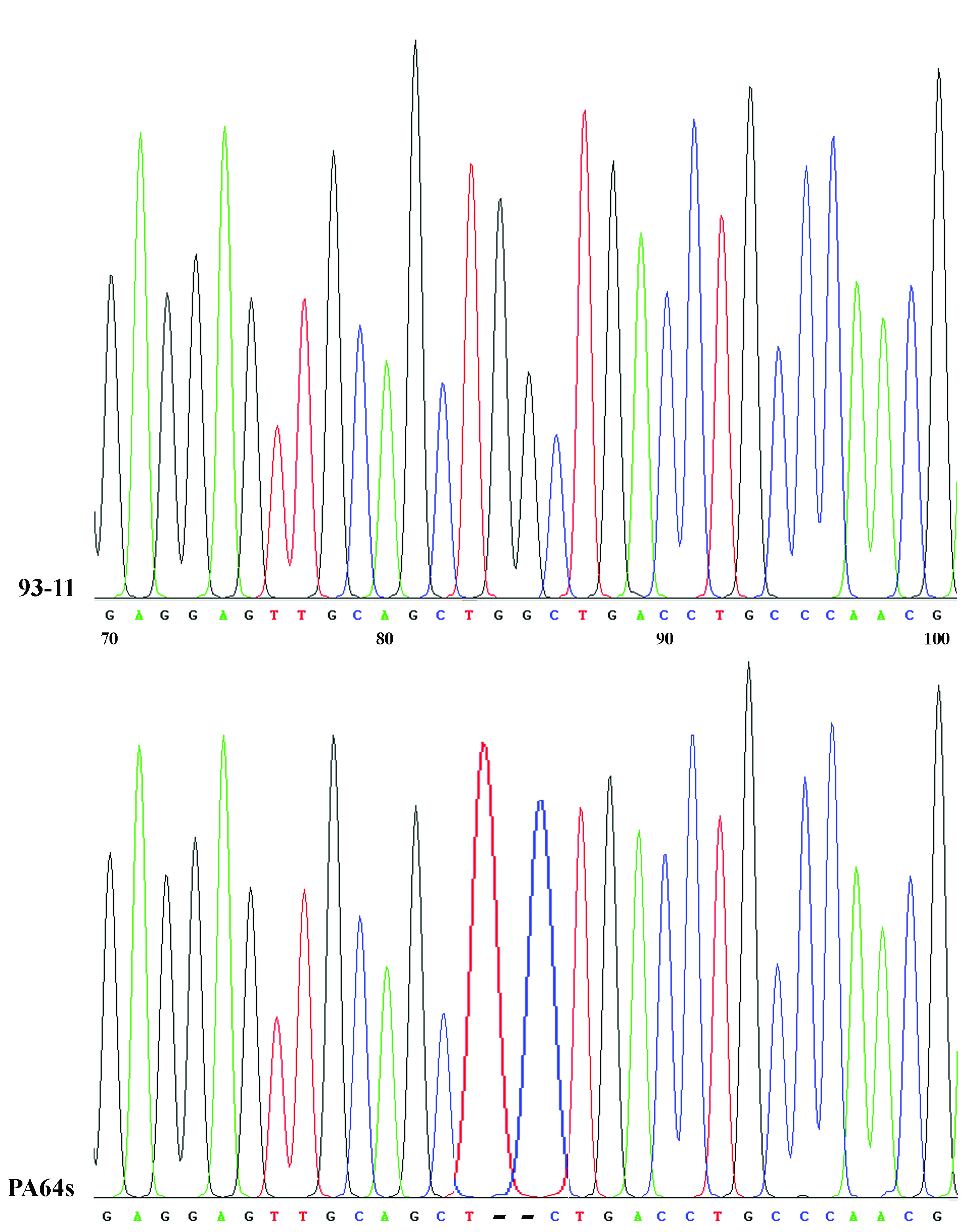

Supplement: FIGURE S2 — Sequence difference of LOC_Os10g04900 between 93-11 and PA64s. [file Image_2.TIF]

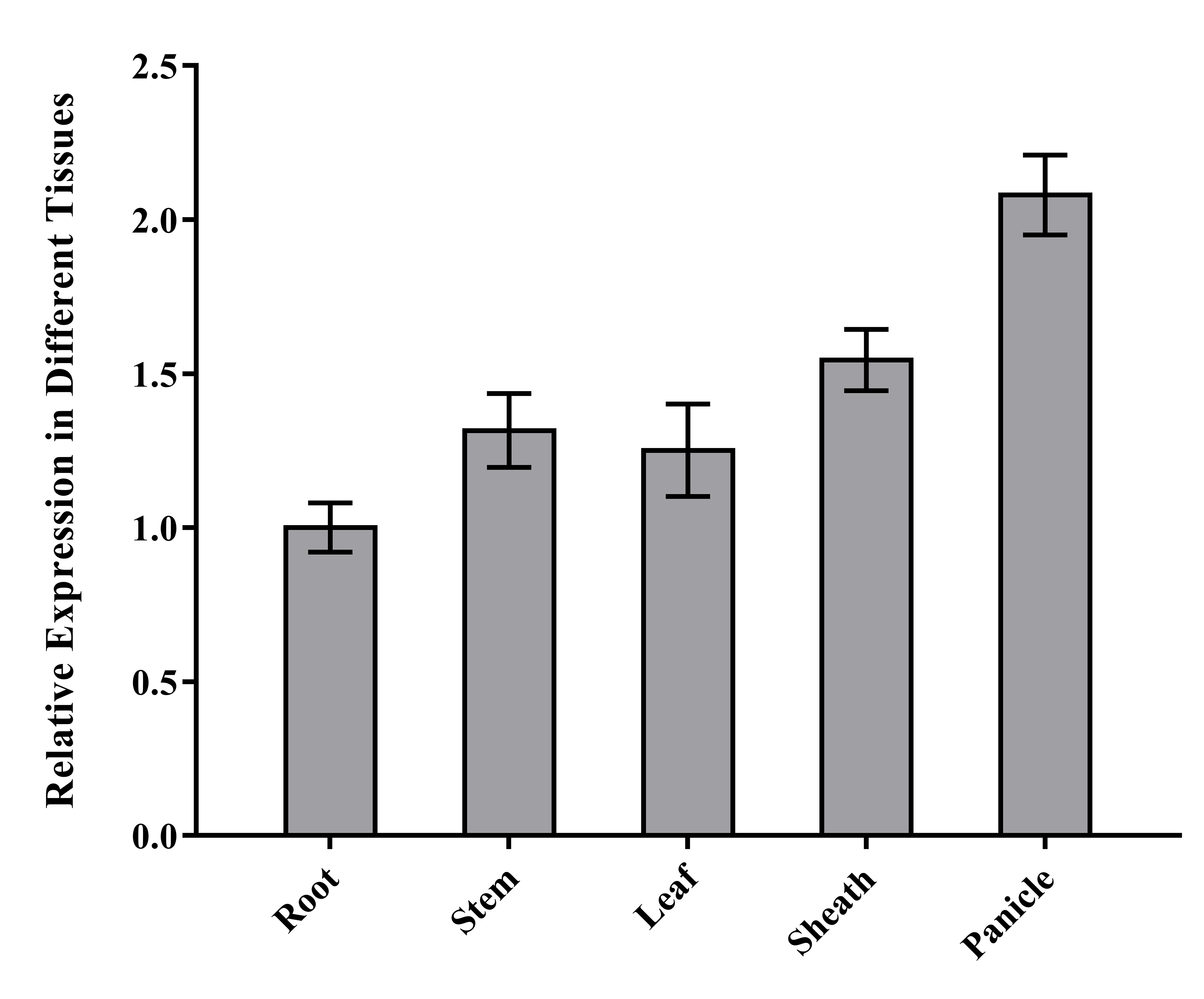

Supplement: FIGURE S3 — Relative Expression of LOC_Os10g04900 in different tissues of PA64s. The error bar for each value represents mean ± SD (n = 3). [file Image_3.TIF]
