# Supplementary material for: Genetic Analysis for Cooking and Eating Quality of Super Rice and Fine Mapping of a Novel Locus qGC10 for Gel Consistency
Source: Front Plant Sci. 2020 Mar 24;11:342. doi: 10.3389/fpls.2020.00342 (PMC7105826; doi:10.3389/fpls.2020.00342)
Supplement: TABLE S1 — Primers for fine mapping of qGC10. [file Table_1.DOC]

**Supplementary Table S1** Primers for fine mapping of *qGC10*

| Marker | Forward primer (5’→3’) | Reverse primer (5’→3’) |
| --- | --- | --- |
| IND-1 | TTTTGCTGAAAGTCGTCGGT | AGCTACATGACTCACTTTGTTCT |
| IND-2 | AGTTTCCATGCAGACACCAG | AAAACAGTCTGCAGTGGTGT |
| IND-3 | AGAGGATGGACACAGCCAAC | GAGATGAACCGGCTGAAGAG |
| IND-4 | GAGGATTCCATGATGCCGTAG | TCCTTATCTTCATTGCCCTCT |
| SNP-1 | TTAGTAGCTTGATATAGGCTTATGTTG | ATGTGGGCAATGCTAGAAAGTC |
| SNP-2 | AGCCATCCAATGTCATCAAG | TCATGATTTGGATAAATAATTTAAGAG |
| IND-5 | TTGGGATGAGGAATGGG | AAGCCAACGAAGACCTG |
| IND-6 | GAAGAGAACTGATGGGAGAGAAGG | GGGACCTACCAAGCGTGTTTT |
